# Historical Differences in Retirement Adjustment in Germany

**DOI:** 10.1093/geroni/igaa057.1499

**Published:** 2020-12-16

**Authors:** Georg Henning, Oliver Huxhold

**Affiliations:** 1 German Centre of Gerontology, Berlin, Germany; 2 German Centre of Gerontology, Berlin, Berlin, Germany

## Abstract

The nature of retirement has been constantly changing over the last decades. Retirement transitions of later-born cohorts differ from those of earlier-born cohorts in terms of sociocultural context and timing. In addition, today’s retirees differ from those who retired earlier in historical time, for example with respect to gender composition, social resources and health status. Gerontological research has rarely addressed the question whether such developments translate into historical differences in retirement adjustment quality. In the current study, we investigated historical differences in perceived retirement adjustment. We distinguished developments for blue-collar and white-collar workers to detect potentially increasing social inequalities. Our pre-registered analyses were based on data from four waves of the German Ageing Survey. The sample included n = 990 participants interviewed either 1996, 2002, 2008 or 2014, who retired in a five-year period before the respective interview (1991-1996, 1997-2002, 2003-2008 and 2009-2014, respectively). Retirement adjustment was measured with three self-report items. Our preliminary results, based on multi-group structural equation modeling, do not provide evidence for a linear improvement or decline of retirement adjustment quality over historical time. White-collar worker reported better adjustment, but this effect was constant over historical time. Our results do not support ideas of fundamental historical differences or growing social inequalities in the individual experience of retirement.

